# Outpatient alcoholism treatment – 24-month outcome and predictors of outcome

**DOI:** 10.1186/1747-597X-4-15

**Published:** 2009-06-29

**Authors:** Michael Soyka, Peggy Schmidt

**Affiliations:** 1 Private Hospital Meiringen, PO Box 612, CH-3860 Meiringen, Switzerland; 2 Psychiatric Hospital, Ludwig-Maximilian-University, Nußbaumstr. 7, 80336 Munich, Germany

## Abstract

**Objectives:**

To study the value of demographic and alcohol-related variables for predicting 24-month treatment outcome in an outpatient setting.

**Methods:**

Prospective observational study with 92 alcohol-dependent patients. Assessments were made by personal interviews at the beginning and end of therapy, and at the 24-month follow-up. Univariate and logistic regression analyses were performed.

*Results: *The mean age was 46.0 (SD = 9.9) years. There were 58 males (65.2%) and 31 females (34.8%). Of the 67 patients interviewed at 2-year follow-up, 58% were abstinent and 79% improved. Differences between abstainers and non-abstainers were found for number of previous detoxifications, and number of patients attempted suicides. In addition, female gender and a higher number of prior treatments predicted negative treatment outcome.

**Conclusion:**

Matching patients to different types of treatment by means of empirically based characteristics may help to improve outcome but research has failed to establish reliable predictors in that area. Data from this follow-up study confirm the role of certain clinical outcome predictors. Additionally, results give further evidence for outpatient treatment as an effective setting for alcohol-dependent patients as indicated by a favourable retention rate (84%) and outcome (minimum abstinence rate 44%).

## Background

Setting and gender effects play a substantial role in treatment of alcoholism. Variables that may predict treatment outcome are of great relevance for optimal allocation of patients to different treatment settings [1-5]. In Germany and other European countries in recent years highly structured outpatient treatment programs have been developed and partially replaced longer inpatient treatment as indicated by a larger number of patients in outpatient clinics but few follow-up studies have been published on the efficacy of these treatments [6]. Variables that were found to be predictive in inpatient treatment [7] do not necessarily have to be so in other treatment settings.

Favourable 3-year outcome results for an intensive alcohol outpatient treatment programme were found in an earlier study [8]. Furthermore, this study identified female gender, number of previous treatments, relapse during treatment, duration of relapse during treatment, treatment drop out and attempted suicides as risk factors for a negative treatment outcome. Identification of predictors should allow improvement of treatment outcome and allocation of patients to the most suitable treatment setting as well as a reduction in the number of treatment failures. A number of different variables are being discussed as potential predictors of treatment outcome. Besides biological parameters such as the GABRA2 genotype [9], the following variables were generated in a literature review.

- Demographics and social functioning measures: employment, gender, socioeconomic status/income, religion [8,10-26],

- Substance-related measures: baseline alcohol consumption, dependence severity, treatment history, alcohol-related self-efficacy, motivation, treatment goal, duration of problem drinking/alcohol dependence, baseline alcohol consumption, craving, [10-13,17,27],

- Other clinical measures: psychopathology rating, neuropsychological functioning [10-14,28-35].

Identification of reliable outcome predictors should help to improve patient allocation and consequently the utilization of resources. Hence the main objective of this study is to evaluate the value of demographic, alcohol-related variables, and psychopathology-related variables for predicting 24-month treatment outcome. In this study we tried to confirm previous research concerning predictors of outcome in an elaborated outpatient treatment setting [8].

According to the previous results we will examine the hypothesis that female gender and treatment drop out are the strongest predictors for relapsing after treatment.

## Methods

### Study design

This was a prospective observational study. The methodology and research instruments were basically the same as those used in a previous study performed in the same treatment setting [8]. The study was conducted at the outpatient facility "Client-oriented Problem Advice Centre Dachau", near Munich. This centre offers a highly structured, intensive, two-phase treatment model. Treatment starts with a three-month motivational phase immediately after detoxification. This phase includes a detailed medical/neurological and psycho-diagnostic examination. Patients are seen on several days per week. They attend a weekly group therapy session and four individual psychotherapy/medical sessions. The motivational phase is followed by an 8-month rehabilitation phase which is the object of research. The therapy concept is integrative and eclectic, and includes psychoanalytical as well as behavioural approaches and methods (three weekly sessions). It is an intensive abstinence-oriented program which was described in detail by Bottlender and Soyka [36].

From January to December 2003, 92 alcohol-dependent patients were consecutively recruited at the start of the outpatient rehabilitation. This was all of the patients (100%) which fulfilled the inclusion criteria defined by health care providers; no formal screening took place. Most patients referred by (family) physician or employer. Patients fulfilled the DSM-IV [37] criteria for alcohol dependence. A further inclusion criterion was a stable residential situation. Exclusion criteria were dependence of benodiazepines and/or illicit drugs, severe physical illness, severe mental disorders and mental disorders requiring inpatient treatment (acute suicidality, psychosis). All patients who entered treatment participated in the study. All patients gave written informed consent to participate in the study.

### Assessment

Both diagnoses and variables relevant for the analyses were recorded in structured face-to-face interviews. Interviews used the European Addiction Severity Index (EuropASI: German version) [38] and were conducted with each patient at the start of the programme (Baseline, T0), at discharge from the treatment unit (T1) and at the 24-month follow-up (T4). The baseline assessment included demographics, past and current psychiatric, medical and substance use-related problems, and drinking parameters. According treatment history the patients were asked about prior detoxification, prior alcohol rehabilitation, and prior treatments for psychiatric problems except for alcoholism. The variable 'suicide attempts' means the lifetime suicide attempts before T0. At discharge the length of time spent in the programme, mode of discharge from the programme (e.g. successfully completed the programme, left prematurely by choice, etc.) and relapses during treatment were recorded. The interviewers were trained psychologists, physicians and medical students and were not involved in the treatment of interviewed subjects; the project coordinator was not a member of the clinical staff.

At T1, patients completed the self-rating Obsessive Compulsive Drinking Scale (OCDS) [[39], German version: [40]] and at T0 the self-rating Beck Depression Inventory (BDI) [41] and the state scale of the State-Trait-Anxiety Inventory (STAI) [42].

Furthermore, abstinence was checked by breathalyser during the entire treatment period as well as at every visit after treatment. In table 1 variables, assessment instruments and assessment times are summarized.

**Table 1:** Variables, assessment instruments and assessment times

T0	T1	T2	T3	T4
Treatment start	At discharge	6 months after discharge	12 months after discharge	24 months after discharge
			
EuropASI/patient files: Demographics Psychiatric, medical and substance use problems Drinking parameters	EuropASI/patient files: Time in treatment Mode of discharge Relapses	Not relevant for this study		Total abstinence during the 24 months "Improved" Relapse
BDI	OCDS			
STAI				

### Definition of outcome criteria

*Abstinence *two years after discharge from treatment was the primary outcome criterion. Abstinence was defined as no subjective report or objective indication of alcohol consumption since discharge from treatment. This criterion was used in the data analyses as dependent variable.

Moreover, in the outcome description the number of patients who completed treatment and the number of improved patients were recorded. 'Improved' was defined according to the classification by Feuerlein and Küfner [7] as less than 30 g (female) or 60 g (male) of alcohol per day, no signs of physical or mental consequences of alcohol abuse or of any pathological drinking pattern, or no more than three drinking periods lasting less than a week (lapses) since discharge from treatment; *'relapse' *was defined as more than three lapses or regular consumption of more than 30/60 g alcohol per day, newly appeared alcohol-related disorders and/or alcohol inpatient treatments.

Furthermore, patients who personally interviewed at the 24-month follow-up were named 'responder'. Non-responders are patients who missed the 24-month follow-up interview. Regarding the sample size, we performed both, the analyse limited to responders vs. analyse including non-responders as relapsers. No significant difference resulted.

### Data analyses

Statistical analyses were performed using SPSS for Windows [43]. Absolute and relative frequencies, means and standard deviations (SD) were calculated for data description. Abstinent and non-abstinent patients as well as responders and non-responders were compared univariat with Chi^2 ^by Pearson (alternative and categorial data), Mann-Whitney-U-test (ordinal data), Kolmogorov-Smirnov-test (metric data) and with backward stepwise logistic regression analyses. In account of the small sample size and the lot of variables we would like to integrate in the analyses, we followed the three steps for model building described by Hosmer and Lemeshow [44] to identify meaningful predictors of outcome. The model is useful for modelling of complex data sets. The process began with univariate analysis for checking potential predictors (Step 1). Step 2 was the manual selection of variables for the multivariate analysis. According to Mickey and Greenland [45] variables whose univariate p-value < 0.25 were candidates for the multivariate analyse. In a third step the importance of each variable integrated in the model was verified and we obtained a preliminary main effects model. This third step included a manual selection of the most important predictor of each category (demographics, substance-related variables and other clinical measures).

*Independent variables *included in the analyses were:

- *Demographics: *age, gender, education (school and professional qualifications), employment status, living circumstances, marital status and socioeconomic status/income (kind of income),

- *Substance-related variables *onset of alcohol use, onset for problem drinking, onset of alcohol dependence, baseline alcohol consumption, craving (OCDS-score), dependence severity (EuropASI) and treatment history,

- *psychopathology-related variables (other clinical measures): *attempted suicide, psychopathology rating (EuropASI), prior psychiatric treatment and symptoms of depression or anxiety (scores of BDI and STAI).

All statistical tests were two-tailed. A p-value of less than 0.05 was considered to be statistically significant. Regarding the sample size, we performed both, the analyse limited to responders vs. analyse including non-responders as relapsers. No significant difference resulted.

## Results

Of the 92 patients enrolled in the study, 77 (83.7%) completed the full outpatient treatment. Two male and one female patient became seriously ill (apoplexy, cerebral haemorrhage, laryngeal carcinoma) and were excluded from further analyses (3 of 92). Data from 67 patients (75.3% of 89) were available for analysis 24 months after discharge: The other 22 patients (24.7%) did not take part in the 24-month follow-up because they declined further participation (n = 13; 14.6%), their new address was unknown (despite a search by the registration office: n = 6; 6.7%) or they could not be contacted despite several attempts (n = 3; 3.4%). Of the interviewed sample (n = 67), 58.2% patients (n = 39) were abstinent and 79.1% patients (n = 53) were abstinent or improved at the 24-month follow-up. If all patients without follow-up data were assumed to be relapsers, 43.8% patients (39 of 89) were abstinent and 59.6% (53 of 89) were abstinent or improved.

### Patients' characteristics and results of the univariate comparison of abstainers and non-abstainers (T4)

The study sample consisted of 58 males (65.2%) and 31 females (34.8%). At admission, the mean age of the patients was 46.0 (SD = 9.9) years. Patients were socially well integrated: Many were married (47.2%), lived together with a partner and children (55.0%) and were employed (75.3%). Further demographic variables are shown in table 2.

**Table 2:** Differences in demographic variables (T0) between abstinent and non-abstinent patients at 24-month follow-up (T4)

	Total sample	Patients responded T4 (n = 67)		Differences abstinent vs. non-abstinent
				
	(n = 89)	abstinent T4 (n = 39)	non-abstinent T4 (n = 28)	
	
Age (M, SD)	46.0 (9.9)	47.0 (9.2)	46.1 (1.6)	n.s.
				
Gender (n, %)				
Male	58 (65.2)	26 (66.6)	15 (53.6)	Pearson Chi^2 ^= 0.84^a^; df = 1
Female	31 (34.8)	13 (33.3)	13 (46.3)	
				
Without secondary school qualifications (n, %)	1 (1.1)	1 (2.6)	0	n.s.
Without professional training (n, %)	14 (15.7)	5 (12.8)	4 (14.3)	n.s.
				
Livelihood (n, %)				n.s.
Gainful employment	67 (75.3)	34 (87.2)	18 (64.3)	
Unemployment benefit	7 (7.8)	1 (2.6)	3 (10.7)	
Pension	7 (7.8)	2 (5.2)	4 (14.3)	
Support by relatives	5 (5.6)	1 (2.6)	2 (7.1)	
Other	3 (3.3)	1 (2.6)	1 (3.6)	
				
Residential situation – living (n, %):				n.s.
alone	33 (37.0)	14 (35.9)	6 (21.4)	
with parents	1 (1.1)	0	0	
with children	5 (5.6)	1 (2.6)	3 (10.7)	
with cohabitant and with/without children	49 (55.0)	24 (61.5)	18 (64.3)	
with friends	1 (1.1)	0	1 (3.6)	
				
Marital status (n, %)				n.s.
Single	18 (20.2)	7 (17.9)	4 (14.3)	
Married	42 (47.2)	20 (51.3)	15 (53.6)	
Separated	9 (10.1)	2 (5.1)	2 (7.1)	
Divorced	17 (19.1)	9 (23.1)	5 (17.9)	
Widowed	3 (3.4)	1 (2.6)	2 (7.1)	

^a^p < 0.25, variable was included in the main effects model.

Alcohol-related as well as psychopathology-related variables are shown in table 3. The average duration of alcohol dependence was 13.6 (SD = 9.3) years and the mean age of onset of alcohol dependence 32.1 years (SD = 10.4). Abstainers and non-abstainers differed in the number of previous detoxifications. On average, the non-abstainers had participated in more alcohol detoxifications (4.7; SD = 7.5) than the abstainers (2.5; SD = 4.9).

**Table 3:** Differences in alcohol-related, treatment-related and psychopathology-related variables (T0/T1) between abstinent and non-abstinent patients 24 months after end of treatment (T4)

	abstinent T4 (n = 39)	non-abstinent T4 (n = 28)	Differences abstinent vs. non-abstinent
			
Age of onset of alcohol use (years: M, SD)	14.4 (3.2)	14.4 (3.0)	n.s.
Age of onset of regular alcohol use (years: M, SD)	21.1 (6.2)	21.8 (7.3)	n.s.
Age of onset of alcohol dependence (years: M, SD)	32.0 (9.9)	32.2 (11.6)	n.s.
Duration of alcohol dependence (years: M, SD)	15.0 (10.6)	13.2 (9.4)	n.s.
Daily alcohol intake (g/day: M, SD)	193.2 (113.5)	157.8 (90.3)	n.s.
			
Number of previous treatments (M, SD) for			
alcohol			
detoxification	2.5 (4.9)	4.7 (7.5)	Mann-Whitney-U = 346.5^*,b^
rehabilitation	1.6 (8.0)	0.4 (0.8)	n.s.
mental health problems	0.4 (0.6)	1.3 (2.3)	Mann-Whitney-U = 411.5^a,b^
somatic problems	2.7 (2.4)	3.5 (2.7)	Mann-Whitney-U = 432.0^a,b^
			
EuropASI Composite Score Medical status	0.18 (0.27)	0.21 (0.33)	n.s.
EuropASI Composite Score Economic situation	0.21 (0.34)	0.33 (0.44)	n.s.
EuropASI Composite Score Employment	0.18 (0.26)	0.16 (0.24)	n.s.
EuropASI Composite Score Alcohol use	0.24 (0.07)	0.26 (0.12)	n.s.
EuropASI Composite Score Drug use	0.00 (0.00)	0.01 (0.03)	n.s.
EuropASI Composite Score Law	0.03 (0.09)	0.00 (0.00)	n.s.
EuropASI Composite Score Family relationship	0.12 (0.19)	0.17 (0.24)	n.s.
EuropASI Composite Score Social relationship	0.08 (0.16)	0.05 (0.12)	n.s.
EuropASI Composite Score Psychiatric status	0.06 (0.09)	0.10 (0.14)	n.s.
			
OCDS total (M, SD)^c^	5.5 (5.5)	7.8 (7.0)	Mann-Whitney-U = 277.5^a^
			
Treatment drop out (n, %)^c^	2 (5.2)	4 (14.3)	Pearson Chi^2 ^= 1.5^a^; df = 1
Single relapse during treatment (n, %)^c^	1 (2.6)	1 (3.6)	n.s.
Repeated relapse during treatment (n, %)^c^	0	2 (7.2)	Pearson Chi^2 ^= 2.7^a^; df = 1
			
Attempted suicide (n, %)	3 (7.7)	7 (25.0)	Pearson Chi^2 ^= 3.5^*,b^; df = 1
STAI (M, SD)	36.9 (7.3)	38.3 (7.9)	n.s.
BDI (M, SD)	7.0 (7.1)	8.9 (6.0)	Mann-Whitney-U = 301.5^a^.

*p < 0.05;^a^p < 0.25; ^b ^variable was included in the main effects model; ^c ^evaluated at T1.

Furthermore, abstainers and non-abstainers differed in attempted suicides until T0: 25% of the non-abstinent patients had attempted at least one suicide, while this relation for the abstainers was 7.7%. No significant differences were found in the results of STAI, BDI or OCDS.

With reference to the three steps for model building, we selected the following variables with an univariate p-value less than 0.25 as candidates for the multivariate analyse: gender, number of previous detoxifications, number of prev. mental health problems, number of prev. somatic problems, treatment drop out, repeated relapse during treatment, attempted suicide, the BDI score, and the OCDS total score.

### Predictors of outcome after 24 months (T4)

In the next step the importance of each variable included in the model was verified and we obtained a preliminary main effects model. The variables 'gender', 'number of previous treatments' and 'attempted suicide' were included in the main effect model. Table 4 presents the final logistic regression model with three significant predictors: gender (OR = 0.2; 95%CI = 0.0–1.0; p < 0.05), number of prior detoxifications (OR = 0.7; 95%CI = 0.6–1.0; p < 0.05) and prior treatments for mental problems (OR = 0.2; 95%CI = 0.1–0.7; p < 0.05).

**Table 4:** Differences between abstinent and non-abstinent patients 24 months after end of treatment (T4) – results of logistic regression analyses

	abstinent T4 (n = 39)	non-abstinent T4 (n = 28)	OR	Wald/df	95%CI
	
Gender (n, %)			0.2*	3.9/1	0.0–1.0
Male	26 (66.6)	15 (53.6)			
Female	13 (33.3)	13 (46.3)			
					
Number of previous treatments (M, SD)					
Detoxification	2.5 (4.9)	4.7 (7.5)	0.7*	3.7/1	0.6–1.0
For mental health problems	0.4 (0.6)	1.3 (2.3)	0.2*	3.9/1	0.1–0.7

*p < 0.05.

### Differences between patients with response or non-response at T4

The groups differed in the retention rate (Pearson Chi^2 ^= 8.1; df = 1; p < 0.01) and number of single (Pearson Chi^2 ^= 4.3; df = 1; p < 0.05) and repeated relapses during treatment (Pearson Chi^2 ^= 11.3; df = 1; p < 0.01). More patients with no response had dropped out (33.3% vs. 9.0%), had a single relapse (14.8% vs. 3.0%) or had repeated relapses (25.9% vs. 3.0%) during the outpatient treatment.

## Discussion

Associations between demographic and clinical variables and outcome in outpatient alcohol treatment were examined in a 2-year follow-up study. The overall treatment results of the 24-month follow-up were in replication of former results comparatively good, with a retention rate over the 8-month treatment phase of 84% and a minimum abstinence rate of 44% (all patients lost to follow-up regarded as relapsers), and in line with previous findings [8]. Of patients personally interviewed at follow-up 57% were abstinent and 21% improved. These results give further evidence for the effectiveness of this outpatient treatment for alcohol-dependent patients [cp. [6,8,36]].

The analyses identified female gender, number of prior detoxifications and prior treatments for mental problems as predictors of negative outcome. In addition, univariate analyses showed effects of suicide attempts. More of the non-abstinent patients had a history of attempted suicides.

These findings will be discussed in the light of previous studies on this subject.

### Demographics

In a quantitative and qualitative review of alcohol treatment research, Jarvis [16]) found gender differences varied as a function of time after treatment. During the first year after treatment, women had a slightly superior treatment outcome; however, this result had reversed one year after treatment. In a review of 38 alcohol outcome studies, Toneatto et al. [17] reported a better treatment outcome of women in 58% of all studies reviewed and no gender differences in the remaining 42%. The Project MATCH Research Group [19] and McKay et al. [18] found better treatment outcome in females, but other studies found no gender differences [e.g. [20,21]]. Reasons for variation in gender effects found in various studies include the different definitions of relapse or a variation in the outcome criteria, statistical methodology, prospective versus retrospective design and sample characteristics [11]. The same result like in the actual study, a less favourable outcome of woman, was reported by Bottlender and Soyka [8] and Anton et al. [22]. It seems that women have other treatment needs than men. This is one result of a study by Grella et al. [26]. They found differences in the treatment needs of women and men [26]. It is possible that different coping strategies are a reason for different demands in alcohol treatment. Sigmond et al. [25] detected the use of different coping strategies by women and men. Additionally, women were found to show different patterns of alcohol exposure and a different course of the disease [e.g. [18,21], and [23]]. Special treatment settings such as outpatient treatment may in some sequences not meet the needs of some female patients. One feasible reason is that female patients more often have an alcoholic spouse compared to male participants [8]. In summary, an understanding of the differences in the treatment needs of women and men seems to be helpful for the development and provision of the most effective alcoholism treatment.

### Alcohol-related variables

Unlike Diehl et al. [20] and others, we did not find any predictive value of the duration of alcohol dependence. The same is true for years of problem drinking, drinks per drinking day [11,29] and age of onset for problem drinking [13]. However, we found that the number of previous detoxifications predicted outcome and further studies showed that there is an association between this number and the alcohol severity. These findings are in line with the previous research indication prior treatment(s) to be a negative predictor [7,10].

### Psychopathology-related variables

In general, psychopathology and psychiatric comorbidity is one of the most robust predictors of outcome in alcohol treatment [14,28]. In our sample, patients who relapsed during the 24-month period had more prior treatments for mental problems and more attempted suicides than the abstinent group. Furthermore previous treatments for mental health problems were an significant outcome predictor. In a previous sample, Bottlender and Soyka [8] also identified the number of previous (alcoholism) treatments and the attempted suicides as risk factors for a negative treatment outcome.

Data on depression and outcome in alcoholism are mixed. Greenfield et al. [30] used the BDI to analyse the relation between time to first drink and current depressive symptoms for 40 women and 61 men participating in an inpatient alcoholism treatment programme. They found no predictive value of depressive symptoms. In addition, Bradizza et al. [34] investigated associations between relapse to alcohol and depressive symptoms and found no relationship between depressive symptomatology measured by BDI and resumption of alcohol use or relapse in patients one year after discharge from inpatient treatment. Like Greenfield et al. [30] and Bradizza et al. [34], we also found no evidence that depressive symptoms (measured with the BDI) have impact on treatment outcome after 24 months. The same applied to anxiety symptoms assessed by STAI. A potential reason for the lack of a relationship between depressive and anxiety symptoms and relapse is the severity of the symptoms, as reflected by the scores: at admission, the scores of STAI and of BDI ranged in the lower to middle range; the patients' scores were not scattered over such a wide range that a clear differentiation would be possible.

In summary, depressive symptoms measured with the BDI and anxiety symptoms measured with the STAI did not predict treatment outcome in a less severely affected sample of patients. Nevertheless, the psychiatric status is not irrelevant as the relapsed patients were treated more frequently for mental disorders.

According the a priori hypothesis:

Female gender was one of the predictors of a negative treatment outcome: treatment outcome was triggered by gender, number of previous detoxifications and of previous treatments for mental problems. A recent systematic review also showed gender to be predictive as was severity of dependence and baseline alcohol consumption [10].

Surprisingly, treatment drop out was not a predictor of relapsing after treatment. The role of previous treatments is very interesting according the allocation to the most suitable current kind of treatment. In a further study we aim to investigate the allocation to three kinds of treatments.

Our study has some limitations. The selection procedure of patients was done before study start. Patients were participants of an outpatient treatment programme and had a stabile residential situation, a rather good level of social adjustment, as indicated by the fairly low unemployment rate, among others. The sample size was rather small; differences may have been larger if all patients had participated in the 24-month follow-up. Still the rate of patients personally interviewed after 2 years was fairly good. Finally we did not integrate a control group.

Matching patients to different types of treatment on the basis may help to improve outcome but research has failed to establish reliable predictors in that area [19]. In general, social variables have a high predictive value [46]. Our data are in line with these findings.

## Conclusion

Despite the limitations which reduce generalizability, the study indicates that alcohol outpatient treatment is an effective treatment option at least in socially more stable patients. Data from this follow-up study confirm the role of certain clinical outcome predictors. Female patients and patients treated more frequently for mental problems were more likely to have a poor 24-month outcome. These findings are basically in line with results of a previous follow-up study [8]. Future research may especially focus on setting and gender effects to improve allocation of patients to different treatment settings.

## Competing interests

The authors declare that they have no competing interests.

## Authors' contributions

MS conceived of the study, participated in its design and coordination and helped to draft the manuscript. PS performed the statistical analysis and drafted the manuscript. All authors read and approved the final manuscript.
